# A Comprehensive Mapping of HIV-1 Genotypes in Various Risk Groups and Regions across China Based on a Nationwide Molecular Epidemiologic Survey

**DOI:** 10.1371/journal.pone.0047289

**Published:** 2012-10-08

**Authors:** Xiang He, Hui Xing, Yuhua Ruan, Kunxue Hong, Chunlin Cheng, Yuanyuan Hu, Ruolei Xin, Jing Wei, Yi Feng, Jenny H. Hsi, Yutaka Takebe, Yiming Shao, Group for HIV Molecular Epidemiologic Survey

**Affiliations:** 1 State Key Laboratory for Infectious Disease Prevention and Control, National Center for AIDS/STD Control and Prevention, China Centers for Disease Control, Beijing, China; 2 AIDS Research Center, National Institute of Infectious Diseases, Tokyo, Japan; Shanghai Medical College, Fudan University, China

## Abstract

**Background:**

China is experiencing a dynamic HIV/AIDS epidemic. While serology based surveillance systems have reported the spread of HIV/AIDS, detailed tracking of its transmission in populations and regions is not possible without mapping it at the molecular level. We therefore conducted a nationwide molecular epidemiology survey across the country.

**Methods:**

HIV-1 genotypes were determined from 1,408 HIV-positive persons newly diagnosed in 2006. The prevalence of each genotype was estimated by weighting the genotype’s prevalence from each province- and risk-specific subpopulation with the number of reported cases in the corresponding subgroups in that year.

**Results:**

CRF07_BC (35.5%), CRF01_AE (27.6%), CRF08_BC (20.1%), and subtype B' (9.6%) were the four main HIV-1 strains in China. CRF07_BC and CRF08_BC were the primary drivers of infection among injecting drug users in northeastern and southeastern China, respectively, and subtype B' remained dominant among former plasma donors in central China. In contrast, all four strains occurred in significant proportions among heterosexuals nationwide, pointing to an expansion of the HIV-1 epidemic from high-risk populations into the general population. CRF01_AE also replaced subtype B as the principal driver of infection among men-who-have-sex-with-men.

**Conclusions:**

Our study provides the first comprehensive baseline data on the diversity and characteristics of HIV/AIDS epidemic in China, reflecting unique region- and risk group-specific transmission dynamics. The results provide information critical for designing effective prevention measures against HIV transmission.

## Introduction

Since the first HIV/AIDS case in China was reported in 1985 [1], an estimated 740,000 individuals have contracted HIV in China by the end of 2009 [2]. In the last quarter century, the main drivers of China’s HIV epidemic have shifted considerably, from blood transmission to sexual transmission. The proportions of injecting drug users (IDUs), heterosexuals, and men who have sex with men (MSM) among HIV positive persons have changed from 44.2%, 11.3% and 0.3% in 1985 - 2005 to 25.8%, 55.7% and 8.6% in 2009, respectively [2].

China has experienced several waves of HIV-1 epidemics since its onset in the late 1980s. In 1989, the first epidemic was among IDUs in Yunnan province, on China’s southwest border near Myanmar [3], [4]. Subtype B’ (Thailand’s variant of subtype B) [3] and subtype C were almost concurrently transmitted to Yunnan from Thailand and India, respectively [5], later generating two new B/C circulating recombinant forms, CRF07_BC and CRF08_BC, most likely in Yunnan [6]–[8]. CRF07_BC spread to northwestern China along a drug traffic route in around 1993 [8]–[10], and CRF08_BC spread eastward to the southern coastal provinces, Guangxi and Guangdong, in around 1990 [6], [8]. IDUs constituted about 70% of HIV cases reported in early 1990s [11]. However, in the early-mid 1990s, unregulated and unsanitary commercial plasma collection in central China resulted in an explosion of HIV-1 subtype B’ among plasma donors in Henan [12] and then dispersed to other provinces in central China [13]–[15]. On the other hand, HIV-1 CRF01_AE was first detected among heterosexuals and IDUs in Guangdong, Guangxi and Yunnan around 1996–1997 [16], [17], and soon spread through sexual routes to other provinces along the southeast coast [18], [19]. More recently, HIV infections among MSM increased rapidly [2]. Besides subtype B of US-European origin, CRF01_AE and CRF07_BC were also identified in MSM populations [20]–[23].

As HIV-1 transmission patterns in China diversify, there is increasing need for detailed, comprehensive analysis on the geographic and demographic distribution of viral genotypes. In this study, we estimated the likely prevalence and distribution of HIV-1 genotypes in China, using more than 1,500 specimens collected throughout China in conjunction with HIV case report data for various risk groups in the respective provinces. Our results provide the first comprehensive dataset on the characteristics and diversity of China’s HIV/AIDS epidemic as related to regional and risk group-specific transmission patterns.

## Methods

### Study Population and Sample Collection

A total of 1,513 plasma specimens were collected from HIV-positive individuals newly diagnosed in 2006 from various risk groups in 30 of the 31 provinces of mainland China except Hainan (Table 1). Plasma samples were collected during routine follow-up visits at local Centers for Disease Control in 2006 - 2008. The study was conducted in a cross-sectional method using stratified random sampling by province. The sampling ratio [numbers of samples collected (b) over numbers of HIV case reports (a)] for each province is shown in Table 1 (column b/a). For provinces with fewer reported cases, higher sampling ratios were used to assure statistical confidence; the median sampling ratios for provinces with <200, 200–499, 500–999, 1,000–3,999, and >4000 cases were 22.1%, 16.4%, 9.5%, 2.9% and 2.1%, respectively (Table 1). No HIV-2 infections were identified in this study. The study was approved by the institutional review boards of the National Center for AIDS/STD Control and Prevention. Written informed consent was obtained from all study participants.

**Table 1:** HIV case report in 2006 and sample collection.

Region	Province	No. of reportedcases (a)	No. of collectedsamples (b)	Sampling ratio(collected) % (b/a)	No. of genotypedsamples (c)	Sampling ratio(genotyped) % (c/a)	Corrected No. of reported cases represented in the study (d)*	representation inthe study % (c/d)
**Northeastern**	**Heilongjiang**	106	23	21.7	23	21.7	91	25.3
	**Jilin**	130	38	29.2	38	29.2	122	32.0
	**Liaoning**	188	40	21.3	40	21.3	179	22.3
**Eastern**	**Beijing**	482	46	9.5	42	8.7	462	9.1
	**Fujian**	160	17	10.6	17	10.6	123	13.8
	**Guangdong**	4551	87	1.9	72	1.6	4484	1.6
	**Hainan**	71	0	0.0	0	0.0	0	0.0
	**Hebei**	134	30	22.4	28	20.9	124	22.8
	**Jiangsu**	301	65	21.6	63	20.9	293	21.5
	**Shandong**	273	60	22.0	55	20.1	268	20.5
	**Shanghai**	694	59	8.5	59	8.5	685	8.6
	**Tianjin**	65	10	15.4	10	15.4	54	18.5
	**Zhejiang**	490	74	15.1	72	14.7	476	15.1
**Central**	**Anhui**	426	65	15.3	60	14.1	369	16.3
	**Henan**	1519	51	3.4	43	2.8	1391	3.1
	**Hubei**	320	56	17.5	55	17.2	319	17.2
	**Hunan**	1048	59	5.6	54	5.2	962	5.6
	**Jiangxi**	222	49	22.1	44	19.8	220	20.0
	**Shanxi**	239	23	9.6	14	5.9	170	8.2
**Northwestern**	**Gansu**	57	12	21.1	9	15.8	44	20.5
	**Neimenggu**	46	13	28.3	7	15.2	42	17.1
	**Ningxia**	39	12	30.8	8	20.5	36	22.9
	**Qinghai**	15	3	20.0	3	20.0	15	31.3
	**Shaanxi**	91	32	35.2	21	23.1	85	24.7
	**Xinjiang**	6018	167	2.8	155	2.6	5977	2.6
	**Xizang (Tibet)**	1	1	100.0	1	100.0	1	100.0
**Southwestern**	**Chongqing**	865	90	10.4	87	10.1	865	10.1
	**Guangxi**	5636	112	2.0	110	2.0	4937	2.2
	**Guizhou**	1037	25	2.4	23	2.2	978	2.3
	**Sichuan**	2978	27	0.9	24	0.8	2540	0.9
	**Yunnan**	7965	167	2.1	167	2.1	7825	2.1
	**Total**	36167	1513	4.2	1404	3.9	34137	4.1

* see Table S1. (d) indicates the summation of the reported cases in the risk groups where the samples for genotyping were available. No. of reported cases in the risk groups from which the samples were not available were excluded from the analysis in this study.

**Figure 1:**
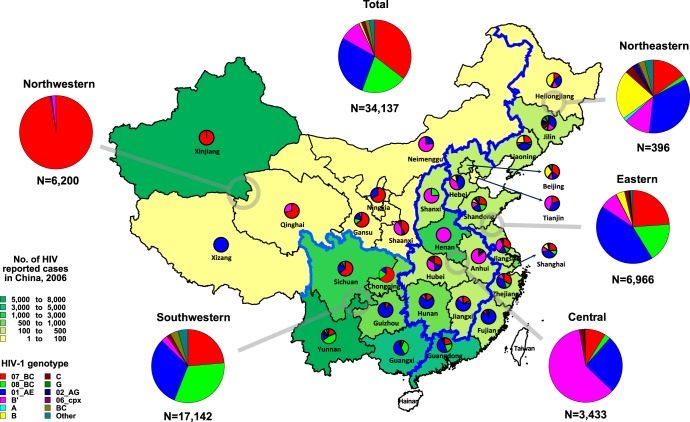
Estimated distribution of HIV-1 genotypes in different provinces in China. Distribution of HIV-1 genotypes in each provinces across mainland China (n = 30), except Hainan, was illustrated based on the dataset tabulated in Table S2.

The study subjects represent all known major risk groups for HIV infection in China. The risk groups include heterosexuals (n = 705), MSM (n = 137), IDU (n = 446), former plasma donors (FPD) (n = 80), blood transfusion (BT) recipients (n = 34), mother to child transmission (MCT) (n = 22), other (n = 6) and unknown (n = 83). The 30 provinces are grouped into four regions (Table 1) according to their proximity and socio-economic status as per guidelines from the National Bureau of Statistics of China [24], [25]: Northeastern (n = 101); Eastern (n = 448); Central (n = 303); and Western (n = 661) regions. In our analysis, we further divided the Western region into Northwestern (n = 240) and Southwestern (n = 421) sub-regions. The boundaries of the respective regions are depicted in Fig. 1.

### Sequence Analysis and Subtype Determination

HIV-1 nucleotide sequences of 1.1-kb *gag* (HXB2∶781–1836 nt) and 540-bp *env* (HXB2∶7002–7541 nt) regions were PCR-amplified and sequenced as described by Cheng *et al* [26]. HIV-1 genotypes were determined based on neighbor-joining tree analysis in comparison with Los Alamos 2010 HIV-1 subtyping references (http://www.hiv.lanl.gov). Phylogenetic analysis was performed using MEGA4 software with bootstrapping of 1,000 replications [27]. The recombinants were analyzed and confirmed with Simplot version 3.5.1 [28]. The HIV-1 genotype of each patient was assigned based on the genotypes of both the *gag* and *env* genes; if only one gene region was available, the genotype of that region was assigned. Samples with different genotypic identification assigned to the *gag* and *env* regions were deemed discordant, and labeled by the *gag*/*env* genotype designations (e.g. CRF01_AE/B).

### Data Stratification and Calculation

Since the transmission of HIV, and therefore the distribution of genotypes, is strongly associated with the geographic location and risk group status of infective individuals [29], we defined the basic unit of our analysis (a subgroup) as each particular risk population in a given province. We defined the *proportion* of an HIV-1 genotype within each subgroup as its proportion among the samples obtained for each subgroup. We then determined the estimated *number* of individuals for each genotype in a subgroup by multiplying the calculated proportion with the total number of HIV cases reported in the subgroup. For a subgroup in which no specimen was obtained, the number of reported cases was removed from the 2006 reported total. The adjusted totals and corresponding sampling ratios for each province is listed in the last two columns [(d) and (c/d)] of Table 1. The actual calculation process is shown in Table S1.

The estimated number of individuals infected with each genotype in each risk group nationwide was obtained by summing the subgroups across all provinces (Table 2). Similarly, the estimated proportions and numbers of individuals infected with each genotype in each geographic region and nationwide were obtained by summing up the subgroups accordingly (Table 2 and 3).

**Table 2:** Distribution of HIV-1 genotypes in different risk groups in China*.

HIV-1 genotype	Risk group							Total (%)
	IDU (%)	Heterosexual (%)	MSM (%)	FPD+BT (%)	MCT (%)	Other (%)	N/A (%)	
**CRF07_BC**	8,344 (48.5)[68.8]	2595 (21.9)[21.4]	62(8.8)[0.5]	43 (3.3)[0.4]	54 (18.2)[0.4]	99 (78.0)[0.8]	925 (34.7)[7.6]	12122 (35.5)[100.0]
**CRF08_BC**	4064 (23.6)[59.0]	2082(17.6)[30.2]	4 (0.6)[0.1]	0 (0.0)[0.0]	74 (25.0)[1.1]	9 (7.1)[0.1]	659 (24.7)[9.6]	6892 (20.2)[100.0]
**CRF01_AE**	3649 (21.2)[38.9]	4709 (39.8)[50.2]	392 (55.8)[4.2]	41 (3.1)[0.4]	37 (12.5)[0.4]	3(2.4)[0.0]	557 (20.9)[5.9]	9388 (27.5)[100.0]
**B'**	380(2.2)[11.6]	1373 (11.6)[41.7]	44 (6.3)[1.3]	1214 (92.5)[36.9]	101 (34.1)[3.1]	0 (0.0)[0.0]	177 (6.6)[5.4]	3289 (9.6)[100.0]
**A**	0 (0.0)[0.0]	20 (0.2)[100.0]	0 (0.0)[0.0]	0 (0.0)[0.0]	0 (0.0)[0.0]	0 (0.0)[0.0]	0 (0.0)[0.0]	20 (0.1)[100.0]
**B**	0 (0.0)[0.0]	64 (0.5)[17.9]	151 (21.5)[42.3]	15 (1.1)[4.2]	0 (0.0)[0.0]	16 (12.6)[4.5]	111 (4.2)[31.1]	357 (1.0)[100.0]
**C**	135 (0.8)[25.4]	287 (2.4)[54.0]	0 (0.0)[0.0]	0 (0.0)[0.0]	0 (0.0)[0.0]	0 (0.0)[0.0]	109 (4.1)[20.5]	531 (1.6)[100.0]
**G**	0 (0.0)[0.0]	10 (0.1)[100.0]	0 (0.0)[0.0]	0 (0.0)[0.0]	0 (0.0)[0.0]	0 (0.0)[0.0]	0 (0.0)[0.0]	10 (0.0)[100.0]
**CRF02_AG**	0 (0.0)[0.0]	22 (0.2)[68.8]	0 (0.0)[0.0]	0 (0.0)[0.0]	0 (0.0)[0.0]	0 (0.0)[0.0]	10 (0.4)[31.3]	32 (0.1)[100.0]
**CRF06_cpx**	0 (0.0)[0.0]	3 (0.0)[100.0]	0 (0.0)[0.0]	0 (0.0)[0.0]	0 (0.0)[0.0]	0 (0.0)[0.0]	0 (0.0)[0.0]	3 (0.0)[100.0]
**BC**	322 (1.9)[51.0]	236(2.0)[37.4]	0 (0.0)[0.0]	0 (0.0)[0.0]	0 (0.0)[0.0]	0 (0.0)[0.0]	73 (2.7)[11.6]	631 (1.8)[100.0]
**Other discordant** **genotypes**	302(1.8)[34.6]	444 (3.7)[50.9]	49 (7.0)[5.6]	0 (0.0)[0.0]	30 (10.1)[3.4]	0 (0.0)[0.0]	48 (1.8)[5.5]	873 (2.6)[100.0]
**Total**	17196 (100.0)[50.4]	11845 (100.0)[34.7]	702 (100.0)[2.1]	1313 (100.0)[3.8]	296 (100.0)[0.9]	127 (100.0)[0.4]	2669 (100.0)[7.8]	34148 (100.0)[100.0]

* Numbers in parentheses and square brackets indicate the proportion of the HIV-infected in each subgroup as a percentage of the national total for that risk group and genotype, respectively. The estimation was done, assuming that HIV case reports reflect the actual prevalence of HIV infection in the respective risk groups and provinces (see Methods).

**Table 3:** Estimated distribution of HIV-1 genotypes in different risk groups and regions in China.

Region	Risk group	07	08	01	B'	A	B	C	G	02	06	BC	Other discordant genotypes	Total
**North** **eastern**	**IDU**	36	0	0	0	0	0	0	0	0	0	0	0	36
	**Heterosexual**	21	0	49	24	6	22	15	0	9	3	6	9	164
	**MSM**	0	0	57	3	0	39	0	0	0	0	0	6	105
	**FPD+BT**	3	0	2	6	0	0	0	0	0	0	0	0	11
	**Other**	0	0	0	0	0	16	0	0	0	0	0	0	16
	**NA**	0	8	30	14	0	8	0	0	0	0	5	0	65
	**Subtotal**	60	8	138	47	6	85	15	0	9	3	11	15	397
**Eastern**	**IDU**	1142	1031	1500	95	0	0	0	0	0	0	0	7	3775
	**Heterosexual**	311	137	966	254	14	36	138	10	13	0	9	0	1888
	**MSM**	38	4	228	37	0	106	0	0	0	0	0	27	440
	**FPD+BT**	0	0	39	94	0	15	0	0	0	0	0	0	148
	**MCT**	0	14	4	5	0	0	0	0	0	0	0	0	23
	**Other**	0	10	3	0	0	0	0	0	0	0	0	0	13
	**NA**	171	15	259	76	0	104	0	0	10	0	0	48	683
	**Subtotal**	1662	1211	2999	561	14	261	138	10	23	0	9	82	6970
**Central**	**IDU**	163	31	546	62	0	0	5	0	0	0	0	0	807
	**Heterosexual**	93	58	307	736	0	0	28	0	0	0	4	14	1240
	**MSM**	10	0	11	0	0	5	0	0	0	0	0	0	26
	**FPD+BT**	38	0	0	1106	0	0	0	0	0	0	0	0	1144
	**MCT**	0	0	0	96	0	0	0	0	0	0	0	0	96
	**NA**	0	0	16	64	0	0	41	0	0	0	0	0	121
	**Subtotal**	304	89	880	2064	0	5	74	0	0	0	4	14	3434
**North** **western**	**IDU**	4271	0	10	4	0	0	7	0	0	0	0	0	4292
	**Heterosexual**	1099	6	18	69	0	0	0	0	0	0	0	0	1192
	**MSM**	0	0	8	4	0	0	0	0	0	0	0	0	12
	**FPD+BT**	2	0	0	8	0	0	0	0	0	0	0	0	10
	**MCT**	48	0	0	0	0	0	0	0	0	0	0	0	48
	**Other**	99	0	0	0	0	0	0	0	0	0	0	0	99
	**NA**	524	0	0	23	0	0	0	0	0	0	0	0	547
	**Subtotal**	6043	6	36	108	0	0	7	0	0	0	0	0	6200
**South** **western**	**IDU**	2732	3001	1593	219	0	0	123	0	0	0	322	295	8285
	**Heterosexual**	1071	1881	3369	290	0	6	106	0	0	0	217	421	7361
	**MSM**	14	0	88	0	0	0	0	0	0	0	0	16	118
	**MCT**	6	60	33	0	0	0	0	0	0	0	0	30	129
	**NA**	230	636	252	0	0	0	68	0	0	0	68	0	1254
	**Subtotal**	4053	5578	5335	509	0	6	297	0	0	0	607	762	17147

## Results

### Distribution of HIV-1 Genotypes in China

A total of 1,404 HIV-1 genotypes of the 1.1-kb *gag* and/or the 540-bp *env* regions were determined from 1,513 plasma specimens collected from newly diagnosed HIV-positive persons in 2006 (Table 1). 1,290 *gag* genotypes (85.3% of samples) and 1,186 *env* genotypes (78.4%) were obtained, and both *gag* and *env* genotypes were available for 1,072 samples (70.9%). There appears to be no significant bias in the success rates of genotyping due to preferred amplification of particular genotypes over others. In calculating the prevalence of HIV-1 genotypes, we weighted the sampled prevalences with the size of the corresponding risk group in any given province; if no specimen was obtained for the risk- and province-specific subgroup, the case count for that subgroup was removed from the analysis (Methods, also see Table S1). This removed 5.6% (2,030 of 36,167) of all reported cases from the national total [(d) in Table 1].

A total of ten major HIV-1 subtypes and circulating recombinant forms, including subtypes A, B, B’, C, and G, and CRF01_AE, CRF02_AG, CRF06_cpx, CRF07_BC, and CRF08_BC, were detected in the study (Table 2). CRF07_BC, CRF01_AE, CRF08_BC, and subtype B’ were the four predominant HIV-1 subtypes circulating in China, accounting for 35.5%, 27.5%, 20.2%, and 9.6% of all infections, respectively (Table 2). These four subtypes alone constituted 92.8% of reported HIV infections in China that year. Minor HIV-1 genotypes included subtypes A (0.06%), B (1.05%), C (1.55%), G (0.03%), CRF02_AG (0.09%), and CRF06_cpx (0.01%). The remaining 4.4% of the samples showed genotype discordance between *gag* and *env* regions: BC (1.84%); BC/CRF01_AE (1.06%); CRF01_AE/C (0.65%); CRF01_AE/BC (0.40%); CRF01_AE/B (0.27%); C/CRF01_AE (0.17%). In the following analysis, we defined the most prevalent discordant genotype (BC) as the eleventh genotype designation, and all other discordant genotypes (2.6%) as the twelfth (Table 2).

### HIV-1 Genotype Distribution by Risk Group

As summarized in Table 2, IDUs and heterosexuals were the largest risk populations, which accounted for 50.4% and 34.7% of estimated HIV infections in 2006, respectively, followed by FPDs and BT recipients (combined) (FPD+BT) (3.8%), MSM (2.1%), MCT (0.9%), and other (0.4%) and unknown risk groups (7.8%).

CRF07_BC (48.5%), CRF08_BC (23.6%) and CRF01_AE (21.2%) were the three main HIV-1 subtypes circulating among IDUs, accounting for 93.3% of infections in this risk group (Table 2). Genotypic diversity was the greatest among heterosexuals, in which all twelve HIV-1 genotype categories were detected (Table 2). The most prevalent genotypes among heterosexuals were CRF01_AE (39.8%), CRF07_BC (21.9%), CRF08_BC (17.6%), and subtype B’ (11.6%). These 4 HIV-1 subtypes accounted for 90.8% of estimated HIV-1 infections among heterosexuals (Table 2). Of note, among MSM where HIV infections are surging recently in China [2], the proportion of CRF01_AE (55.8%) exceeded than that of subtype B of US-European origin (21.5%) that predominated previously. In contrast, subtype B’ predominated among FPD+BT (92.5%) (Table 2). Various HIV-1 strains were detected among MCT: B’ (34.1%), CRF08_BC (25.0%), CRF07_BC (18.2%), and CRF01_AE (12.5%), which may reflect the trends among heterosexuals.

### HIV-1 Genotype Distribution by Geographic Region

As summarized in Table 3, HIV-1 infections were the most concentrated in the western region [(6,200+17,147) of 34,148, 68.4%], followed by eastern (6,970 of 34,148, 20.4%), central (3,434 of 34,148, 10.1%), and northeastern (397 of 34,148, 1.2%).

Figure 1 illustrates the estimated region- and province-specific distribution of HIV-1 genotypes (also see Tables 3 and S2). In the northeastern region, heterosexuals (164 of 397, 41.3%) and MSM (105 of 397, 26.4%) were two major risk groups, CRF01_AE (138 of 397, 34.8%), B (85 of 397, 21.4%), CRF07_BC (60 of 397, 15.1%) and B' (47 of 397, 11.8%) were the predominant genotypes (Table 3). The high proportion of MSM (26.4%) was a unique feature in this region. Consistent with the high concentration of MSM, subtype B was detected at a higher proportion (21.4%) in the northeast than any other regions, although CRF01_AE infections were now more prevalent among MSM than subtype B (54.3% and 37.1%, respectively; see Table 3).

In the eastern region, IDUs (3,775 of 6,970, 54.2%) and heterosexuals (1,888 of 6,970, 27.1%) were the main risk groups (Table 3), and CRF01_AE (2,999 of 6,970, 43.0%), CRF07_BC (1,662 of 6,970, 23.8%) and CRF08_BC (1,211 of 6,970, 17.4%) were major circulating strains. This proportion of genotypes likely reflects the predominance of IDUs and heterosexuals in this region.

In the central region, the main risk groups of HIV infections were heterosexuals (1,240 of 3434, 36.1%), FPD/BT (1,144 of 3,434, 33.3%), and IDUs (807 of 3,434, 23.5%) (Table 3). The dominance of FPD+BT was unique to this region. Subtype B’ showed the highest prevalence (2,064 of 3,434, 60.1%), followed by CRF01_AE (880 of 3,434, 25.6%), CRF07_BC (304 of 3,434, 8.9%) and CRF08_BC (89 of 3,434, 2.6%) (Table 3). While subtype B’ predominated among FPD+BT (1,106 of 1,144, 96.7%), it was also the most prevalent strain among heterosexuals (736 of 1,240, 59.4%), followed by CRF01_AE (307 of 1,240, 24.8%).

In the northwestern region, IDUs (4,292 of 6,200, 69.2%) were the most important risk group, followed by heterosexuals (1,192 of 6,200, 19.2%). CRF07_BC predominated (6,043 of 6,200, 97.5%) in this region as a whole, and was highly prevalent both among IDUs and heterosexuals: 99.5% (4,271 of 4,292) and 92.2% (1,099 of 1,192), respectively (Table 3).

In the southwestern region, IDUs (8,285 of 17,147, 48.3%) and heterosexuals (7,361 of 17,147, 42.9%) were the two main risk groups, contributing almost equally to the epidemic. CRF08_BC (5,578 of 17,147, 32.5%), CRF01_AE (5,335 of 17,147, 31.1%), and CRF07_BC (4,053 of 17,147, 23.6%) were three major circulating strains. Of note, BC (607 of 17,147, 3.5%) and other discordant genotypes (762 of 17,147, 4.4%) were detected at significant proportions in this region. They were mostly from Yunnan [(496+439) of 7,826, 11.9%], Sichuan (230 of 2,540, 9.1%) and Guangxi provinces [(105+87) of 4,936, 3.9%] (Table S2). This is the region where we observed the highest viral diversity after the eastern region (Table S2).

### The Distribution of China’s Four Major HIV-1 Strains by Region and Risk Group

**Figure 2:**
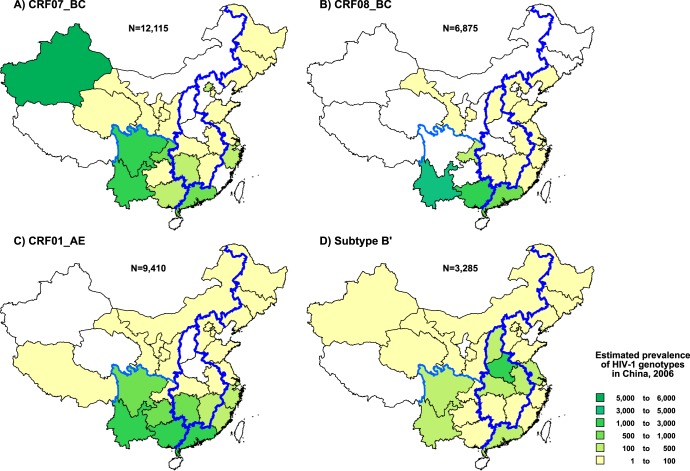
Estimated geographic distribution of the four major HIV-1 genotypes in China. Prevalence of the four major HIV-1 genotypes, including CRF07_BC (A), CRF08_AE (B), CRF01_AE (C) and subtype B' (D), is estimated for each province by the methods described in Methods. Corrected prevalence is color-coded as indicated in the inset.

Figure 2 illustrates the geographic distributions of the four major HIV-1 strains (CRF07_BC, CRF08_BC, CRF01_AE, and subtype B') that accounted for 92.8% of infections in China (also see Table 2).

CRF07_BC was detected in 24 of the 30 provinces (except Fujian, Hebei, Henan, Neimenggu, Shanxi, and Xizang [Tibet]), accounting for an estimated 35.5% (12,122 of 34,148) of HIV-1 infections in China. Most of CRF07_BC infections (90.0%) were found in the western and southern provinces of China, including Xinjiang (5,946 of 12,122, 49.1%), Sichuan (1,634 of 12,122, 13.5%), Yunnan (1,451 of 12,122, 12.0%) and Guangdong (998 of 12,122, 8.2%) (Table S2, Fig. 2A). IDUs and heterosexuals were the two main risk groups for the spread of CRF07_BC, accounting for 68.8% (8,344 of 12,122) and 21.4% (2,595 of 12,122), respectively (Table 2).

While CRF08_BC was identified in 16 provinces in China (Fig. 2B, Table S2), 92.7% of CRF08_BC infections were detected in three southern provinces: Yunnan (3,644 of 6,892, 52.9%), Guangxi (1,762 of 6,892, 25.6%), and Guangdong (983 of 6,892, 14.3%). IDUs (4,064 of 6,892, 59.0%) and heterosexuals (2,082 of 6,892, 30.2%) were the two major risk groups for CRF08_BC infections (Table 2).

CRF01_AE was identified in 26 provinces (none from Henan, Qinghai, Shanxi, and Xinjiang). A majority (80.8%) of CRF01_AE infections were detected in Guangxi (2,634 of 9,388, 28.1%), Guangdong (2,227 of 9,388, 23.7%), Yunnan (1,204 of 9,388, 12.8%), Guizhou (858 of 9,388, 9.1%), and Hunan (664 of 9,388, 7.1%) (Fig.2C, Table S2). Heterosexuals (4,709 of 9,388, 50.2%) and IDUs (3,649 of 9,388, 38.9%) were the main risk groups of CRF01_AE infections (Table 2).

HIV-1 subtype B’ was identified in 28 provinces (except Ningxia and Xizang [Tibet]) and was the most wide-spread HIV-1 strain in China in terms of geographical reach (Fig. 2D). The highest prevalence of subtype B’ was observed in central and southwestern China, especially in Henan (1,391 of 3,289, 42.3%), Anhui (319 of 3,289, 9.7%) and Yunnan (295 of 3,289, 9.0%) (Table S2). Due to the history of subtype B’ transmission in the early-mid 1990s, FPDs were considered to be a primary risk group infected with subtype B’ [30]. However, as shown in Table 2, the proportion of subtype B’ infections was now highest among heterosexuals (1,373 of 3,289, 41.7%), followed by FPD+BT (1,214 of 3,289, 36.9%) (Table 2). This strongly suggests subtype B’ had spread from FPDs into the general population [30], [31].

## Discussion

We conducted a nationwide cross-sectional study on the HIV-1 genotype distribution in China, combining molecular data with province-specific estimates of the number of HIV infections in each risk population. Our study revealed a high level of complexity of the HIV epidemic in China. We detected a total of 10 HIV-1 subtypes and CRFs (Table 2) found in circulation in all continents of the world. This likely reflects the increasing mobility of people in the country, with over 34 million outbound travelers and 49 million inbound across China’s borders in 2006 [32].

Viral diversity varied considerably in different risk populations and regions in China. Subtype B' predominated among FPD+BT (92.5%) (Table 2), consistent with the region’s history of a single explosive HIV epidemic due to contamination in commercial plasma donations in the early-mid 1990s in central China [30]. Similarly, CRF07_BC predominated among IDUs in northwestern China (99.5%) (Table 3), In contrast, we observed the highest viral diversity among heterosexuals: all 12 genotype categories were detected in this population (Table 2). Furthermore, as typically seen in subtype B’ in central China and CRF07_BC in northwestern China, these two strains were also prevalent among heterosexuals in the respective regions: B’ (59.4%) in central China; CRF07_BC (92.2%) in northwestern China (Table 3). This suggests that the spread of HIV-1 from the particular high-risk populations (IDUs and FPDs) into general populations was significantly facilitated through heterosexual transmission in those regions [30], [31], [33], [34].

As well, differences in viral diversity between geographical regions point to differences in the drivers of HIV-1 transmission. CRF07_BC predominated in the northwestern region in the vast majority of risk groups (over 90%) (Table 3). This was especially true for Xinjiang: CRF07_BC was the single predominant HIV-1 strain even among heterosexuals, a genotypically diverse risk group in other regions of the country. This uniformity indicates a strong regional founder effect in the local HIV epidemic and suggests a lack of incoming transmissions from other regions. In contrast, although subtype B’ was the main HIV genotype (60.1%) in central China, its prevalence was highest in the FPD+BT population, less so in heterosexuals, and was rarely found amongst local IDU and MSM (Table 3). Henan province, the center of the FPD epidemic, was the exception, in which subtype B’ predominated in all risk groups. Infections in all other geographical regions were driven by multiple HIV-1 genotypes, which suggest that with a few exceptions, the impact of geographical communities on HIV transmission was weaker than that of behavioral communities (i.e. risk groups). Interventions for curbing new infections may therefore benefit more from implementing risk behavior-specific measures across geographical boundaries than from a more regionally focused approach.

Further, changes in the relative prevalence of genotypes nationwide and among populations of interest illustrate shifts in China’s HIV transmission patterns. The proportion of subtype B’ amongst all infections has decreased from 47.5% in 1998 and 29.1% in 2002 [35] to 9.6% in 2006, in accordance with national policies that strictly regulated blood donations since the late 1990s [36]. However, in 2006, heterosexual contact has overtaken blood transmission routes as the greatest risk group for subtype B’ infections nationwide (41.7% and 36.9%), and B’ also predominated amongst heterosexual infections in the central region (59.4%; Table 3). At the same time, the prevalence of CRF01_AE infections has increased from 9.6% in 1998 and 15.5% in 2002 [35] to 27.5% in 2006 (Table 2). This is likely related to the increasing predominance of sexual transmission across the country. CRF01_AE was the most prevalent HIV-1 strains among heterosexuals (39.8%) and MSM (55.8%) (Table 2), and was highly represented in all geographic regions except the northwestern and central regions where local genotypes prevailed (Fig. 1). Notably, transmission among MSM was previously predominated by subtype B (of US and European origin) in major cities of China [2], but has now been eclipsed by the increasing prevalence of CRF01_AE. This is consistent with previous observations in MSM population in Beijing [20], Liaoning [21] and Shijiazhuang [22]. These trends underscore the increasing significance of sexual transmission in China’s HIV/AIDS epidemic.

Our study has several limitations. Sampling bias may have occurred due to limited sample size in provinces with low HIV-1 prevalence. Bias may also have been introduced during the adjustment of genotype prevalences for risk- and province-specific subgroups, which excluded some subgroups of very low prevalence from calculation (e.g. IDUs in Anhui; see Table S1). Additionally, risk assessment and attribution are also subject to biases; in particular, some MSM, commercial sex workers, and commercial sex clients may be unwilling to disclose their risk status. Future iterations of the national molecular epidemiologic survey should aim to more adequately sample each province- and risk-specific subgroup and to better assess risk statuses.

In summary, our results illustrate the recent state of China’s HIV/AIDS epidemic, reflecting the country’s unique region- and risk group-specific transmission patterns. Our data provide information critical for designing effective interventions to limit future HIV transmission in China. As current approaches in HIV vaccine development often remain limited in eliciting immune responses against wide-ranging viral subtypes [37], [38], improved knowledge of genotype prevalences can help inform vaccine research and development efforts tailored towards the particular HIV epidemic profile in China and nearby regions. In addition, we propose that our method for estimating genotype prevalence (also see [29]), which links molecular genotyping data with the numbers of HIV case reports in region- and risk-group specific subpopulations, may be widely applied to future molecular epidemiologic analyses. We aim to continue monitoring the trends in HIV-1 subtype distribution in China to identify and characterize newly emerging properties of public health importance.

## Supporting Information

Table S1 **Estimated numbers and proportions of HIV-1 infections for region- and risk-specific subgroups in China.**
(XLS)

Table S2 **Estimated distribution of HIV-1 genotypes in different regions/provinces in China.**
(DOC)
