# Supplementary material for: A Comprehensive Mapping of HIV-1 Genotypes in Various Risk Groups and Regions across China Based on a Nationwide Molecular Epidemiologic Survey
Source: PLoS One. 2012 Oct 8;7(10):e47289. doi: 10.1371/journal.pone.0047289 (PMC3466245; doi:10.1371/journal.pone.0047289)
Supplement: Table S2 — Estimated distribution of HIV-1 genotypes in different regions/provinces in China. (DOC) [file pone.0047289.s002.doc]

**Table S2. Estimated distribution of HIV-1 genotypes in different regions/provinces in China**

| **Region** | **Province** | **07** | **08** | **01** | **B'** | **A** | **B** | **C** | **G** | **02** | **06** | **BC** | **other** | **Total** |
| --- | --- | --- | --- | --- | --- | --- | --- | --- | --- | --- | --- | --- | --- | --- |
| **Northeastern** | **Heilongjiang** | 13 | 0 | 25 | 13 | 3 | 38 | 0 | 0 | 0 | 0 | 0 | 0 | 92 |
|  | **Jilin** | 3 | 0 | 41 | 21 | 3 | 6 | 15 | 0 | 9 | 3 | 11 | 12 | 124 |
|  | **Liaoning** | 44 | 8 | 72 | 13 | 0 | 41 | 0 | 0 | 0 | 0 | 0 | 3 | 181 |
|  | **Subtotal** | 60 | 8 | 138 | 47 | 6 | 85 | 15 | 0 | 9 | 3 | 11 | 15 | 397 |
| **Eastern** | **Beijing** | 160 | 0 | 66 | 46 | 8 | 139 | 0 | 0 | 8 | 0 | 0 | 35 | 462 |
|  | **Fujian** | 0 | 5 | 102 | 8 | 0 | 0 | 8 | 0 | 0 | 0 | 0 | 0 | 123 |
|  | **Guangdong** | 998 | 983 | 2227 | 156 | 0 | 0 | 92 | 0 | 0 | 0 | 0 | 27 | 4483 |
|  | **Hebei** | 0 | 4 | 52 | 50 | 0 | 15 | 4 | 0 | 0 | 0 | 0 | 0 | 125 |
|  | **Jiangsu** | 95 | 33 | 46 | 101 | 6 | 3 | 9 | 0 | 0 | 0 | 0 | 0 | 293 |
|  | **Shandong** | 74 | 48 | 56 | 37 | 0 | 21 | 5 | 0 | 0 | 0 | 9 | 20 | 270 |
|  | **Shanghai** | 167 | 79 | 244 | 80 | 0 | 83 | 20 | 10 | 0 | 0 | 0 | 0 | 683 |
|  | **Tianjin** | 11 | 0 | 19 | 24 | 0 | 0 | 0 | 0 | 0 | 0 | 0 | 0 | 54 |
|  | **Zhejiang** | 157 | 59 | 187 | 59 | 0 | 0 | 0 | 0 | 15 | 0 | 0 | 0 | 477 |
|  | **Subtotal** | 1662 | 1211 | 2999 | 561 | 14 | 261 | 138 | 10 | 23 | 0 | 9 | 82 | 6970 |
| **Central** | **Anhui** | 16 | 16 | 19 | 319 | 0 | 0 | 0 | 0 | 0 | 0 | 0 | 0 | 370 |
|  | **Henan** | 0 | 0 | 0 | 1391 | 0 | 0 | 0 | 0 | 0 | 0 | 0 | 0 | 1391 |
|  | **Hubei** | 97 | 0 | 58 | 116 | 0 | 5 | 41 | 0 | 0 | 0 | 4 | 0 | 321 |
|  | **Hunan** | 151 | 20 | 664 | 84 | 0 | 0 | 28 | 0 | 0 | 0 | 0 | 14 | 961 |
|  | **Jiangxi** | 40 | 11 | 139 | 25 | 0 | 0 | 5 | 0 | 0 | 0 | 0 | 0 | 220 |
|  | **Shanxi** | 0 | 42 | 0 | 129 | 0 | 0 | 0 | 0 | 0 | 0 | 0 | 0 | 171 |
|  | **Subtotal** | 304 | 89 | 880 | 2064 | 0 | 5 | 74 | 0 | 0 | 0 | 4 | 14 | 3434 |
| **Northwestern** | **Gansu** | 28 | 6 | 7 | 3 | 0 | 0 | 0 | 0 | 0 | 0 | 0 | 0 | 44 |
|  | **Neimenggu** | 0 | 0 | 10 | 32 | 0 | 0 | 0 | 0 | 0 | 0 | 0 | 0 | 42 |
|  | **Ningxia** | 24 | 0 | 12 | 0 | 0 | 0 | 0 | 0 | 0 | 0 | 0 | 0 | 36 |
|  | **Qinghai** | 10 | 0 | 0 | 5 | 0 | 0 | 0 | 0 | 0 | 0 | 0 | 0 | 15 |
|  | **Shaanxi** | 35 | 0 | 6 | 37 | 0 | 0 | 7 | 0 | 0 | 0 | 0 | 0 | 85 |
|  | **Xinjiang** | 5946 | 0 | 0 | 31 | 0 | 0 | 0 | 0 | 0 | 0 | 0 | 0 | 5977 |
|  | **Xizang (Tibet)** | 0 | 0 | 1 | 0 | 0 | 0 | 0 | 0 | 0 | 0 | 0 | 0 | 1 |
|  | **Subtotal** | 6043 | 6 | 36 | 108 | 0 | 0 | 7 | 0 | 0 | 0 | 0 | 0 | 6200 |
| **Southwestern** | **Chongqing** | 564 | 172 | 95 | 18 | 0 | 6 | 0 | 0 | 0 | 0 | 6 | 6 | 867 |
|  | **Guangxi** | 314 | 1762 | 2634 | 34 | 0 | 0 | 0 | 0 | 0 | 0 | 105 | 87 | 4936 |
|  | **Guizhou** | 90 | 0 | 858 | 30 | 0 | 0 | 0 | 0 | 0 | 0 | 0 | 0 | 978 |
|  | **Sichuan** | 1634 | 0 | 544 | 132 | 0 | 0 | 0 | 0 | 0 | 0 | 0 | 230 | 2540 |
|  | **Yunnan** | 1451 | 3644 | 1204 | 295 | 0 | 0 | 297 | 0 | 0 | 0 | 496 | 439 | 7826 |
|  | **Subtotal** | 4053 | 5578 | 5335 | 509 | 0 | 6 | 297 | 0 | 0 | 0 | 607 | 762 | 17147 |
|  | **Total** | 12122 | 6892 | 9388 | 3289 | 20 | 357 | 531 | 10 | 32 | 3 | 631 | 873 | 34148 |
